# Pharmacokinetics of maraviroc in plasma and breastmilk in a treatment-experienced perinatally HIV-1-infected woman

**DOI:** 10.1097/QAD.0000000000002360

**Published:** 2019-09-02

**Authors:** Cornelia Feiterna-Sperling, Renate Krüger, Alieu Amara, Saye Khoo, Catriona Waitt

**Affiliations:** a Charité – Universitätsmedizin Berlin, Humboldt-Universität zu Berlin and Berlin Institute of Health, Department of Pediatric Pneumology, Immunology and Intensive Care Medicine, Berlin, Germany; b Department of Molecular and Clinical Pharmacology, University of Liverpool, United Kingdom.

Maraviroc (MVC), a C-C chemokine receptor type five (CCR5) antagonist, was approved as part of combination antiretroviral therapy (cART) in 2007, for use in treatment-experienced adults infected with CCR5-tropic HIV-1 [1]. Whilst, with current treatment strategies, such as maternal cART, low rates of mother-to-child transmission (MTCT) have been reported, in high-income countries, breastfeeding is not recommended because of the potential MTCT risk. However, since 2017, European [2] and United States [3] guidelines have acknowledged that some HIV-infected women may wish to breastfeed, and should be given appropriate support in this decision. Whilst data exist describing the transfer of NRTI, NNRTI, and protease inhibitors to breastfed infants [4,5], the pharmacokinetics and safety of MVC in lactating women and their breastfed infants have not been reported. Here, we present the first case of MVC in a breastfeeding mother.

A 36-year-old perinatally HIV-1-infected woman received MVC (150 mg twice daily), lamivudine (150 mg twice daily) and lopinavir/ritonavir (400/100 mg twice daily). Her plasma HIV-RNA has remained undetectable with a CD4^+^ count above 500 cells/μl on this regimen for over a decade.

In 2018, at 38+4 weeks of gestation, she delivered a healthy girl (2710 g, 49 cm, head circumference 35 cm, APGAR score 10 at 5 min). Standard neonatal chemoprophylaxis with oral zidovudine (4 mg/kg twice daily) for 14 days was given according to German--Austrian guidelines [6]. Although not recommended, breastfeeding was chosen. Exclusive breastfeeding continued until 6 months of age, with complete weaning by 7 months. Clinical and laboratory assessment at 2, 4, and 8 weeks, and 3, 6, 9, and 12 months after birth revealed normal development. Full blood cell count, renal, and liver parameters remained within normal range. HIV-DNA PCR results were consistently negative, and at 12 months of age, an HIV antibody test was negative.

At 5 months postpartum, a 12-h pharmacokinetic sampling of maternal plasma and breastmilk was performed to assess the breastmilk transfer and estimate infant exposure to MVC. After approval by the local ethics committee of the Charité University Medicine Berlin, the mother gave her informed consent. Paired maternal plasma and breastmilk samples were obtained predose (0 h), and 1, 2, 4, 6, 8, and 12 h following observed dosing. Thirteen days later, one single plasma sample from the still exclusively breastfed infant was obtained during a routine follow-up visit. Samples were frozen at −30 °C until shipment on dry ice to the laboratory (University of Liverpool, UK) for analysis.

Plasma concentrations of MVC were determined by validated liquid chromatography tandem mass spectrometry method as previously described [7], with a modification for breastmilk. Briefly, the standards and quality control samples were prepared by spiking known concentration of drugs (TRC, Ontario, Canada) into breastmilk (donated by consenting volunteers through the UK Northwest Milkbank with ethics approval) to obtain a calibration curve (range 2.5–2500 ng/ml). MVC stable isotope was used as internal control to minimize matrix effect.

Interday and intraday precision measured at the quality control levels were 5.60% (3.71–6.72) and 3.04% (2.00–4.40), respectively (mean, range). Interday and intraday accuracy were 0.84% (−7.02–6.99) and 3.30% (−2.78 to 10.61), respectively. Mean recovery at all quality control levels was 98.68% (SD, 5.69%).

The plasma MVC pharmacokinetic profile in the mother (Fig. 1) was comparable with published data for postpartum women [8], with full results reported in the legend to Fig. 1. MVC was undetectable in the single infant sample.

**Fig. 1:**
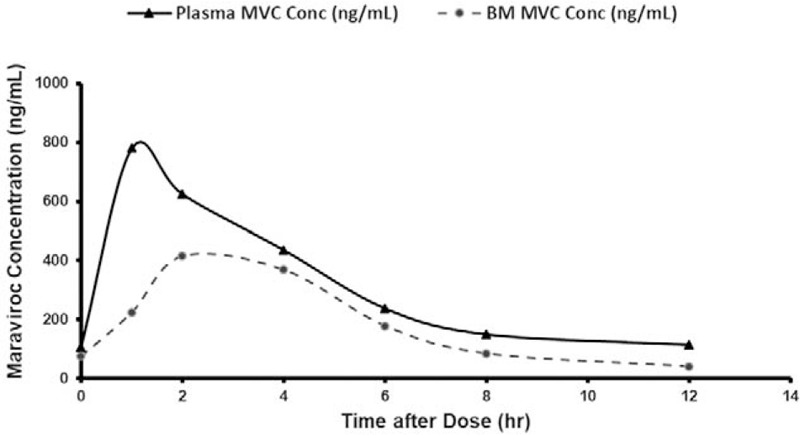
Maraviroc maternal plasma and breastmilk concentration time-profiles at 5 months postpartum.

Maraviroc concentrations in plasma and breastmilk measured by validated liquid chromatography tandem mass spectrometry as described in the case report. The AUC_0–12 h_ for maraviroc in plasma was 3790 h ng/ml with a *C*_max_ of 780 ng/ml reached after 1 h. The breastmilk AUC_0–12 h_ was 2317 h ng/ml with a *C*_max_ of 415 ng/ml reached after 2 h. The milk-to-plasma (M : P) ratio of AUC_0–12_ _h_ was 0.61.

A significant breastmilk transfer of MVC was demonstrated, with a milk-to-plasma (M : P) ratio of 0.61, consistent with studies in lactating rats, which indicated that MVC is extensively secreted into rat milk [9]. MVC is licensed for children (2 years and older) weighing at least 10 kg, at a starting dose of 50 mg. Assuming an infant milk intake of 150 ml/kg/day, we estimate a daily MVC ingestion of less than 2 mg in this 6.5 kg infant. Concerns relating to breastmilk exposure of antiretroviral drugs relate to both infant toxicity and the potential for HIV drug resistance to develop, should MTCT occur in the presence of low drug concentrations. Although MVC was not detected in the infant, it should be noted that the lower limit of quantification of the assay (2.5 ng/ml) is above the IC_90_ (0.57 ng/ml) [10]; low, but clinically relevant infant concentrations were possible. Data from a single case must be interpreted with caution and more data regarding MVC in breastfeeding mother-infant pairs are needed.

In conclusion, there is significant penetration of MVC into breastmilk with a M : P ratio of 0.61, but MVC was undetectable in the breastfed infant.

## Acknowledgements

C.W. is funded by a Wellcome Trust Clinical Postdoctoral Fellowship WT104422MA. S.K. has received funding from ViiV Healthcare, Gilead Sciences, Merck and Janssen for support of the Liverpool HIV drug interactions resource.

### Conflicts of interest

C.F., R.K., A.A., and C.W. have no conflicts of interest.
